# Serological and molecular detection of Toscana and other Phleboviruses in patients and sandflies in Tunisia

**DOI:** 10.1186/s12879-014-0598-9

**Published:** 2014-11-15

**Authors:** Ons Fezaa, Youmna M’ghirbi, Gianni Gori Savellini, Lamia Ammari, Nahed Hogga, Henda Triki, Maria Grazia Cusi, Ali Bouattour

**Affiliations:** Laboratoire d’Epidémiologie et Microbiologie Vétérinaire, Service d’Entomologie Médicale, Institut Pasteur de Tunis, Université Tunis El Manar, Tunis, Tunisia; Department of Medical Biotechnologies, University of Siena, Policlinico Le Scotte, Siena, Italy; Service de Maladies Infectieuses, CHU la Rabta Tunis, Faculté de Médecine Tunis, Université Tunis El Manar, Tunis, Tunisia; Laboratoire de Recherche Diversité Génétique des Entérovirus et Virus des Hépatites, Institut Pasteur de Tunis, Faculté de Médecine de Tunis, Université Tunis El Manar, Tunis, Tunisia

**Keywords:** Toscana virus, ELISA, Nested RT-PCR, Viral isolation, Sandfly Fever Sicilian virus, Punique virus, Phlebovirus, Sandflies, Phlebotomus, Tunisia

## Abstract

**Background:**

Our aim is to detect the infection by Toscana virus (TOSV) and other Phleboviruses in the sera and cerebro-spinal fluid (CSF) of patients with meningitis in Tunisia. We examined various species of phlebotomus present in Tunisia to determine whether or not a direct relationship exists between cases of meningitis and the viruses circulating in the insect vectors.

**Methods:**

Patients with the meningeal syndrome were tested for anti-TOSV IgM and IgG using an indirect Enzyme-Linked Immunosorbent Assay (ELISA) and for the presence of TOSV and other Phleboviruses using a RT-PCR test.

An entomological study was carried out using CDC light traps to trap sandflies in different bioclimatic zones of Tunisia. Collected sandflies were tested by RT-PCR for the presence of TOSV and other Phleboviruses and subsequently by viral isolation on Vero cells.

**Results:**

Of 263 patients were tested using ELISA of which 12.16% (n = 32/263) were IgM positive for anti TOSV. Of these 32 patients, 78% (n = 25/32) were IgG positive. 12.86% (n = 18/140) of the CSF samples tested by RT-PCR were positive for the Toscana virus.

One CSF sample tested by RT-PCR revealed the presence of Sandfly Fever Sicilian Virus (SFSV). The Punique virus was identified in one sandfly pool.

**Conclusions:**

This study confirms, for the first time, that TOSV is involved in a neurological disorder in North Africa. The incidence of this involvement in Tunisia conforms with observations made in other Mediterranean countries. Moreover, for the first time, a molecular approach was used to detect SFSV in a Tunisian patient displaying neurological symptoms.

**Electronic supplementary material:**

The online version of this article (doi:10.1186/s12879-014-0598-9) contains supplementary material, which is available to authorized users.

## Background

The Toscana virus (TOSV) is an arthropodborne phlebovirus of the Bunyaviridae family that is endemic in Mediterranean countries where the insect vectors *Phlebotomus perniciosus* and *P. perfiliewi* are present [1]. TOSV is involved in acute neurological diseases in humans, particularly during the summer. Disease incidence peaks in August, which correlates with the life cycle of the phlebotomus vectors [2] that play a role in transmitting the virus [3].

Reports have been made of TOSV infection associated with aseptic meningitis, meningoencephalitis (AMM), or with encephalitis without meningitis [4],[5]. Some unusual cases of AMM were described in Italy and Spain [6],[7]. Asymptomatic infections and infections that do not affect the central nervous system, such as febrile erythema or influenza-like illnesses, have also been described [8]. Consequently, it is not possible to define a characteristic symptomatology for neurological infections from TOSV [9]. To date, no reinfections have been detected and pre-existing immunity plays a role in limiting the disease in areas where TOSV is prevalent currently [10],[11]. The mean duration of the disease is seven days. The outcome is usually favorable [3].

TOSV has a segmented RNA genome composed of 3 units: L (large), M (medium), and S (small) encoding the viral proteins [12]-[14]. A phylogenetic analysis has demonstrated that TOSV isolates from Spain differ from the original isolates from Italy; two clusters have been identified as lineage A (Italy) and lineage B (Spain) [15]. A new lineage C has been described in both Croatia and Greece [16],[17]. The obtained nucleotide sequence showed a homology that is similar to the TOSV sequences from the A and B lineages (75%-82%) although it clusters with neither.

The well-known difficulty of directly diagnosing acute viral neurological infections is due to a short viremic phase with a low viral load during clinical symptomatology that generally corresponds to the period of hospitalization and clinical sampling [18]. The virus can, however, be isolated from cerebrospinal fluid (CSF) and/or serum if it is collected during the acute phase of the disease. TOSV replicates in Vero, BHK-21, CV-1, and SW13 cells with cytopathic effect [19].

A study conducted in Morocco showed the presence of TOSV in the sandfly vectors; however no data on human epidemiology were published [20]. In Tunisia, the epidemiology of the TOSV infection remains largely unknown, despite the availability of reliable diagnostic laboratory tools and epidemiological surveillance. A few recent studies have reported that 10% of the patients (n = 31) with meningeal syndrome had specific IgM against TOSV [21]. A serological survey using an ELISA test showed a 9.5% seroprevalence of TOSV among healthy individuals [22]. In a recent letter, Bichaud et al. [23] acknowledged finding that two Phleboviruses -- the TOSV and the Punique virus -- co-circulate in sand flies collected in Tunisia but no definitive evidence of a TOSV infection in aseptic meningitis infections was found.

The objective of this work was to detect infection by TOSV and other Phleboviruses in the sera and cerebo-spinal fluid of subjects with meningitis during the summer. We examined various phlebotomus species circulating in Tunisia to prove the existence of a direct relationship between cases of meningitis and virus circulation in the vector.

## Methods

### Sample collection

Between June 2011 and November 2012, 331 patients with meningeal syndrome (symptoms include acute fever, headaches, and the presence of more than 10 CSF white blood cells per mm^3^) from different regions of Tunisia (Tunis in the north, Bizerte to the west, Monastir to the southeast, Sousse along the northeastern coast, Sfax along the southeastern coast and Gabes in the south) were admitted to Tunisian hospitals. Bacterial and fungal cultures of CSF samples tested negative. Suspecting a viral aetiology, the clinical virology laboratory of the Pasteur Institute of Tunis systematically searched for enteroviruses, herpes simplex virus, and West Nile virus, but the analysis proved negative. Samples (sera and CSF) collected from the 331 patients were examined for TOSV or other Phleboviruses infection.

Informed consent from the patient's participants to this study was obtained. This study was approved by The Bioethics Committee of the Institut Pasteur de Tunis.

### Serological investigation

The 191 sera and 72 CSF samples collected were tested in duplicate for the presence of TOSV-specific IgM and IgG antibodies using an indirect in-house ELISA. Wells of polystyrene plates were coated with a predetermined optimum quantity of positive and negative antigens prepared on a Vero cell culture infected by the reference strain ISS Phl3 kindly provided by the Bunyaviridae Laboratory (Professor Bouloy, Institut Pasteur de Paris), in phosphate-buffered saline (PBS) (pH 7.2) and incubated overnight at 4°C. Human sera were diluted at 1:100 in PBS-TM (PBS, 0.05% Tween 20, 3% milk). Each assay included two negative and two positive control sera. Goat anti-human IgM and Goat anti-human IgG (Fc-specific) peroxidase conjugated antibody (Sigma-Aldrich, Germany) was diluted at 1:1000 for sera and at 1/10 for CSF and added to each well. A color reaction was developed by adding a substrate solution containing 3,3′,5,5′-tetramethylbenzidine (TMB) (Sigma-Aldrich, Germany). The reaction mixture was incubated for 75 min. at 37°C at each step, and washed extensively with PBS-Tween. H2SO4 was added at a final concentration of 1 N to stop the reaction. All serum samples were tested in duplicate with viral and negative control antigens [21],[22]. The IgM and IgG ELISA cut-off values were determined by the mean +3 standard deviations of the read optical density. The borderline value was calculated as the mean value of 30 known negative sera plus 3 standard deviations. The OD of each sample was read at a double wavelength of 450 nm and 620 nm .

### Molecular investigation

#### Detection of TOSV and other Phleboviruses in CSF samples

*RNA extraction and RT-PCR:* RNA extraction was performed using the NucleoSpin RNA II kit (Macherey Nagel, Germany) following the manufacturer's instructions. Eluted RNA was collected and stored at -80°C when not immediately used.

RT-PCR and nested PCR were performed using degenerated primer pairs specific for TOSV L genomic segment as described by Sanchez-Seco et al. [24] with some modifications. To avoid any risk of contamination, 6 μL of RNA were used in a one-step RT-PCR reaction with NPhlebo1+/NPhlebo1- primers rather than the two-step protocol described elsewhere. Two different nested PCR reactions were performed in parallel using the degenerated NPhlebo2+/NPhlebo2- primers, suitable for detecting Toscana, Naples, Sicilian, Rift Valley fever, Uukuniemi and Punta Toro viruses, and the Atos2/NPhlebo2+ primers for the specific detection of the TOSV [24]. Several measures were taken to avoid possible contamination, including establishing separate spaces to set up PCRs, filter tips and UV radiations. In addition, tests were run on only a few samples at the same time. Different negative controls (no DNA, uninfected sample and extracted no DNA) were used. A viral extract (culture of TOSV, ISS Phl.3 strain) generously provided by Dr. M.G. Cusi, was used as a positive control.

#### Detection of TOSV and other Phleboviruses in sandflies

**Figure 1:**
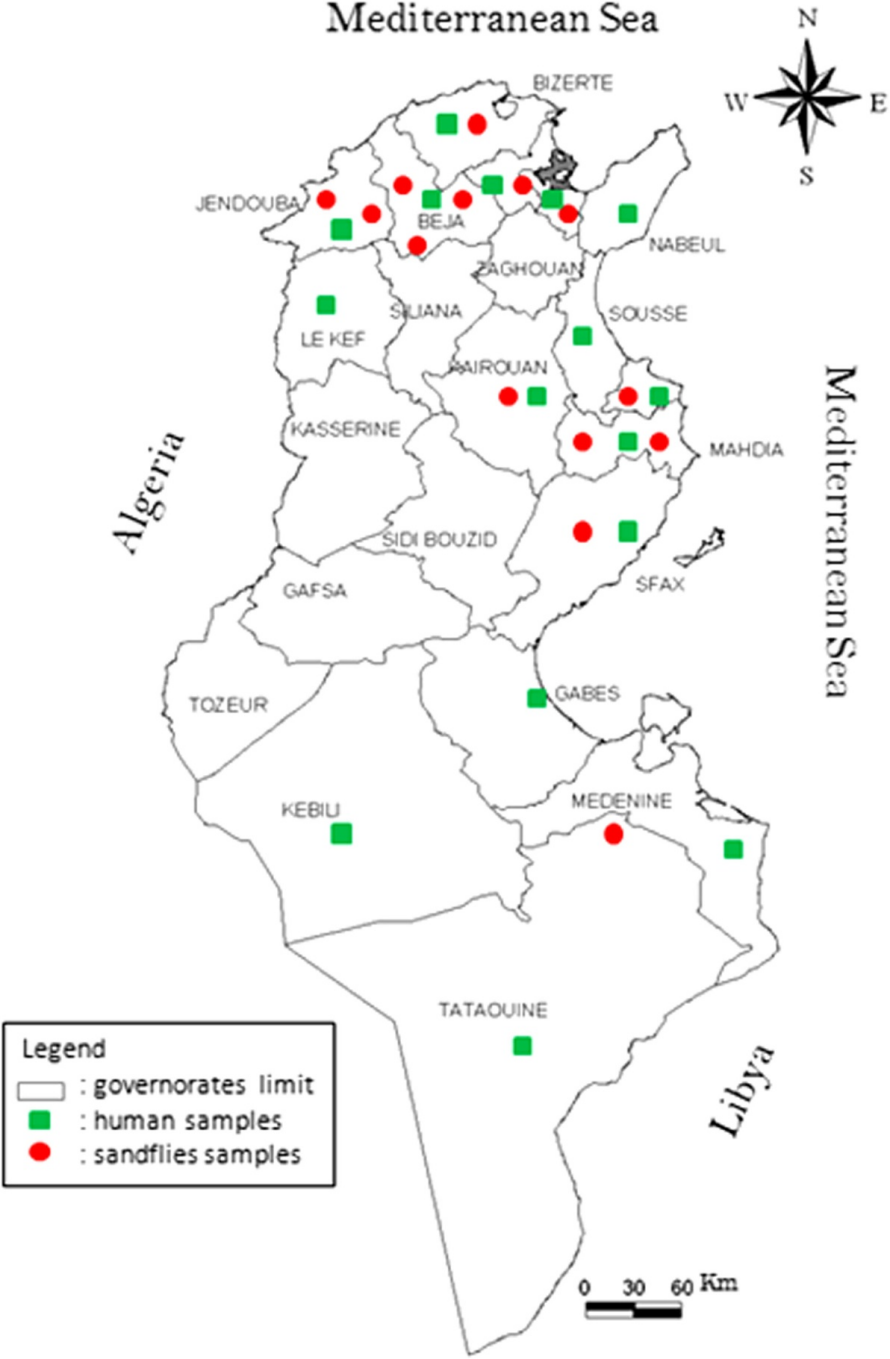
**Map of Tunisia indicating sandflies capture sites and human AMM cases.**

*Capture of phlebotomine sandflies:* Using CDC light traps (John W. Hock Company, Gainesville, FL, USA), phlebotomine sandflies were captured after dusk and until dawn from June to November (2011 and 2012), the period and time when they are active in Tunisia, in the regions where patients showed clinical AMM (Figure 1). Once trapped, the sandflies were immediately stored in liquid nitrogen.

### Pooling and RNA extraction

Stored sandflies were separated into pools of males and females. The males were preserved in alcohol for later identification whereas the females, which are hematophagous, were prepared for viral molecular identification. For this purpose, the head and genitalia of each female were cut for taxonomic diagnosis as per Croset et al. [25], while the thorax (which encloses the salivary glands) and abdomen were used for RNA extraction. Once the taxonomic work had been carried out, pools were made of a maximum of 30 abdomens of females of the same species captured during the same period from the same capture site.

Each pool was ground in 350 μl of 3% fetal bovine serum (FBS, Sigma-Aldrich, Germany) enriched phosphate-buffered saline (PBS). Two hundred μl of ground solution were used for total RNA purification with the RNeasy Plus Mini kit (Qiagen, Germany) as per the manufacturer's instructions. RT-nested PCR was performed to detect TOSV in sandflies as described above.

### Viral isolation

Viral isolation on Vero cells (ATCC CCL-81) was performed on some PCR-positive sandfly pools, as described by Sanchez-Seco et al. [24], with minor modifications: Vero cells were grown in Dulbecco's Modified Eagle Medium (DMEM) (Lonza, Milan, Italy), supplemented with 2% FBS (Lonza, Milan, Italy) and 0.01% penicillin and streptomycin (Lonza, Milan, Italy), as a monolayer in 96 wells plate. Fifty μl of ground sandfly pools were used to infect the monolayers, after which 100 μl of DMEM without FBS were added. Cultures were maintained and monitored daily for 7-15 days until a cytopatic effect (CPE) became evident. Positive cultures were expanded on Vero cells and cell-culture supernatants were stored in aliquots at -80°C.

### Sequencing and data analysis

Specific TOSV detection was carried out by sequencing PCR products as described previously [24]. The PCR products were purified using the ExoSAP cleanup procedure (Amersham Biosciences, France). All nucleotide sequences were obtained using the Big Dye Terminator v.3.1 Cycle Sequencing Kit (Applied Biosystems, Foster City, CA, USA) and the 3130 automated sequencer (Applied Biosystems, Foster City, CA, USA). Sequences were edited and aligned using DNA Baser Sequence Aligner v3.5.4 software (Heracle BioSoft SRL, www.DnaBaser.com) to obtain optimal sequence alignment files. A BLAST analysis at the NCBI database was made to retrieve sets of homologues exhibiting high scores to the L gene of TOSV.

To build phylogenetic tree, the maximum parsimony [26] method calculating bootstrap confidence values of 100 bootstrapping trials, using the MEGA 5.2 software, was used.

### Statistical analyses

Statistical differences among the groups were determined using Pearson's Chi-square test. To calculate the Chi2, the Epi Info v.6.04 was used. A probability (P) value of less than 0.05 was considered statistically significant.

### Nucleotide sequence accession numbers

The sequences generated in this study have been deposited in the DNA Data Bank of Japan (DDBJ) under accession numbers [DDBJ: AB904897 to AB904924 and AB905360 to AB905362].

## Results

### Studied samples

From the 331 patients (1-81 years old), 140 CSF samples (88 men, 52 women) and 191 sera (112 men, 79 women) were tested for Phlebovirus infection. CSF samples (n = 72) were tested by both PCR and enzymatic immunoassay (ELISA) to detect specific IgM. The remaining (n = 68) were tested only by PCR because only limited amount of materials were available from sera reactive individuals. All 191 sera were tested using ELISA.

### IgM/IgG antibodies against TOSV

The presence of TOSV-specific IgM was investigated in the 263 sera and CSF samples collected from patients admitted with AMM to various hospitals in Tunisia. Of these, 12.16% (CI_95%_ [8.47%-16.74%]) tested positive for anti-TOSV IgM: the prevalence of TOSV IgM was detected in 13.6% (26/191) of the serum samples and 8.3% (6/72) of the CSF samples. The different rates detected in sera and CSF samples were not significant (p = 0.24). Geographically, most positive samples came from Tunis (n = 10), Mahdia (n = 7) and Bizerte (n = 6) while the balance (n = 9) came from ten other governorates (Figure 1). The anti-TOSV IgM positive samples were also tested for the presence of IgG antibodies against TOSV. Among them, 25 (78%) were IgG positive. Unfortunately, we were unable to obtain a second serum sample from patients that would have allowed us to follow the antibodies titres.

### Molecular investigation

#### Detection of TOSV and other Phleboviruses in CSF samples

Of the 140 CSF samples tested by RT-PCR, 18 (12.86%, CI_95%_ [7.80%-19.56%]) were positive with both phlebovirus-generic and TOSV-specific primer sets. The TOSV-positive samples (14 men, 4 women) were from Tunis (n = 14), Bizerte (n = 2), Mahdia (n = 1) and Sfax (n = 1). Moreover, one sample was sequenced that had tested positive with the NPhlebo2 pair of primers but negative with the specific TOSV primers Atos2. Of the samples tested, 72 CSF samples were investigated by both RT-PCR and ELISA: two of these samples (2.7%) had antibodies reactive to the TOSV antigen, six of the 72 CSF samples (8.3%) were positive by PCR and negative by ELISA, one (1.3%) was negative by nested PCR and positive by ELISA for anti-TOSV IgM and IgG, while 63 (87.5%) were negative by both methods.

#### Detection of TOSV and other Phleboviruses in sandflies

Between 2011 and 2012, a total of 8040 sandflies were collected by CDC light traps. Of these, 2000 males and 1050 females underwent taxonomic identification that made it possible to diagnose 14 species (Table 1). The remaining sandflies were conserved at -80°C for subsequent studies.

**Table 1:** **Sampling regions, number of tested and TOSV infected female's sandflies**

Region (Bioclimatic zone)	Collected species	Tested species (No.of sandflies tested/No. of pools)	No of positive pools
Bizerte (Sub-humid)	*P. perniciosus*	*P. perniciosus* (75/3)	3
	*P. longicuspis*	*P. longicuspis* (25/1)	0
	*P. papatasi*	*P. papatasi* (25/1)	0
	*P. perfiliewi*	*P. perfiliewi* (50/2)	1
Tunis (Semi arid)	*P. perniciosus*	*P. perniciosus* (75/3)	2
	*P. papatasi*	*P. papatasi* (25/1)	0
	*P. perfiliewi*	*P. perfiliewi* (25/1)	0
	*S. minuta parroti*	*S. minuta parroti* (25/1)	0
Beja (Sub-humid)	*S. minuta parroti*	*P. perniciosus* * (75/3)	2
	*P. perniciosus*		
	*P. perfiliewi*		
	*P. papatasi*	*P. perfiliewi* (25/1)	1
	*S. fallax*		
	*S. antennata*	*P. papatasi* (50/2)	0
	*S.dreyfussi*		
Jendouba (Arid)	*P. papatasi*	*P. papatasi* (30/1)	0
	*P. perniciosus*	*P. perniciosus* (28/1)	0
	*P. longicuspis*	*P. longicuspis* (27/1)	0
	*S. minuta parroti*		
Monastir (Semi arid)	*P. perniciosus*	*P. perniciosus* (30/1)	1
	*P. papatasi*	*P. papatasi* (60/2)	0
	*P. perfiliewi*	*P. perfiliewi* (30/1)	1
	*S. minuta parroti*	*S. minuta parroti* (30/1)	0
	*P. longicuspis*		
	*P. langeroni*		
	*S. dreyfussi*		
	*Pa. alexandri*		
	*S. antennata*		
	*P. ariasi*		
Mahdia (Semi arid)	*P. perniciosus*	*P. perniciosus* (30/1)	0
	*P. papatasi*		
	*P. perfiliewi*	*P. papatasi* (30/1)	0
	*S. minuta parroti*		
	*P. longicuspis*	*P. perfiliewi* (30/1)	0
	*P. langeroni*		
	*S. dreyfussi*	*S. minuta parroti* (30/1)	0
	*Pa. alexandri*		
	*S. antennata*	*S. antennata* (30/1)	0
	*P. ariasi*		
Kairouan (Arid)	*P. perniciosus*	*P. papatasi* (30/1)	0
	*P. papatasi*		
	*P. perfiliewi*		
	*S.minuta parroti*		
	*S.fallax*	*P. perfiliewi* (60/2)	2
	*S.christophersi*		
	*P. longicuspis*		
Sfax (Arid)	*P. pernciciosus*	*P. pernciciosus* (25/1)	0
	*P. papatasi*		
	*P. perfiliewi*		
	*S. dreyfussi*		
	*S. minuta parroti*		
	*Pa. alexandri*	*P. papatasi* (25/1)	0
	*Pa. chabaudi*		
	*S. antennata*		
	*P. longicuspis*		
Tataouine (Saharan)	*P. papatasi*	*P. papatasi* (25/1)	0
	*P. perniciosus*		
	*P. longicuspis*		
	*S. minuta parroti*	*P. longicuspis* (25/1)	0
	*Pa. chabaudi*		
**Total**	14	1050/39	13

*One pool of *P. pernicious* PCR positive for Punique Virus.

The female pools were subjected to RNA extraction for RT-PCR detection of Phleboviruses. Of the 39 pools tested, 14 were positive with phlebovirus-generic primers. Among them, 13 were positive with TOSV-specific primer sets: 8 positive pools of *P. perniciosus* and 5 of *P. perfiliewi*. A single pool tested positive with the NPhlebo2 primers but not with the TOSV specific primers. The positive pools of sandflies had all been collected in 5 governorates located in Tunisia's sub-humid, semi-arid and arid bioclimatic zones (Table 1).

No virus was detected in the other *Phlebotomus* and *Sergentomya* species that were tested.

### Sequencing and culture results

A total of 31 samples (19 CSF samples, 12 sandfly pools) were sequenced using primers specific for Phlebovirus (NPhlebo2) and TOSV (ATos2).

Of the 19 CSF-derived amplicons that were sequenced:

- 15 sequences (from 120-180 sequenced nt) were obtained with Atos2 set primers [DDBJ: AB904897 to AB904911] and three sequences (from 200-250 sequenced nt) were obtained with NPhlebo2 set primers [DDBJ: AB904918, AB904919, AB905360] The BLAST analysis on the National Center for Biotechnology Information website retrieved a unique hit consisting of the TOSV polymerase gene with a 98-99% identity score with French [GenBank: DQ975233, KC700343], 95-97% with Moroccan [GenBank:JN832571] and 95% with Tunisian [GenBank:JX867537] strains of TOSV.
- Only one CSF-derived amplicon sequenced [DDBJ: AB905361] was similar (98%) to the published sequence of the Sandfly Fever Sicilian Virus (SFSV) [GenBank: EF095551] derived from sandflies collected in Algeria [27].

Of the 12 pool-derived amplicons that were sequenced:

- Six sequences [DDBJ: from AB904912 to AB904917] and five sequences were obtained with NPhlebo2 set primers [DDBJ: from AB904920 to AB904924]. The BLAST analysis revealed 98-99% identity with French [GenBank: DQ975233, KC700343] and 95-97% with Moroccan [GenBank: JN832571] TOSV strains.
- In an effort to isolate the virus for more significant sequence information, we randomly selected eight pools, including those that were positive with only the Nphlebo2 primer set, and seeded them onto Vero cells to grow the virus. Only a single sample showed a marked cytopathic effect after eight days. The BLAST of culture-derived PCR product [DDBJ: AB905362] was strongly homologous (95%) with the Punique virus strain PI-B4-2008 [GenBank: JF920133] from Tunisia.

### Phylogenetic analysis

**Figure 2:**
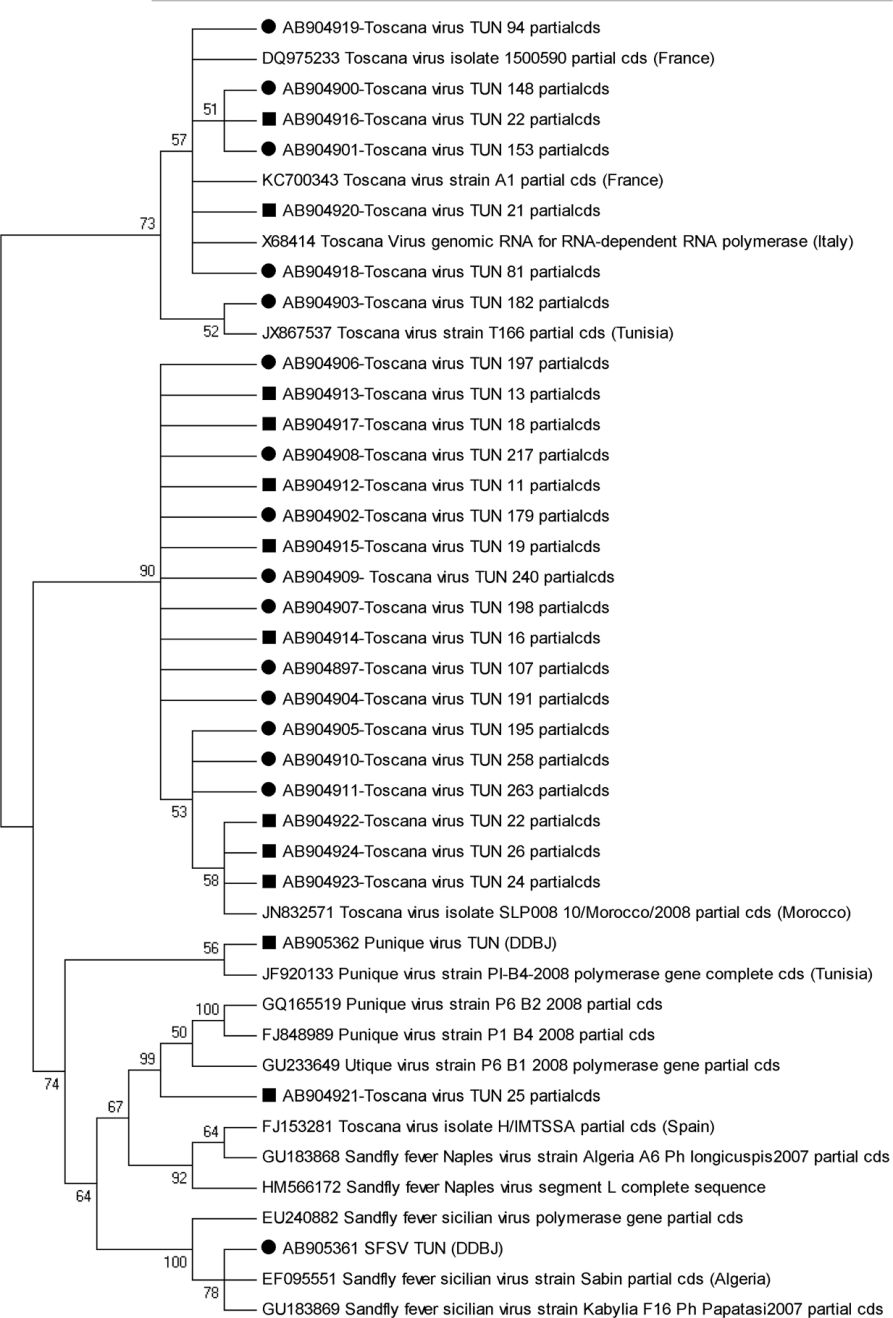
**Phylogenetic analysis of the L segment of Toscana virus (TOSV) isolates from Human CSF (Filled circle) and pools of sandflies (Filled square) collected in Tunisia, and homologous sequences of other selected TOSV stains.** Sequences are identified by DDBJ accession number, and strain name.

In a maximum parsimony analysis based on the alignment of the L segment of TOSV, a phylogenetic tree was constructed from sequences of the TOSV isolate obtained from GenBank (Figure 2) using a maximum parsimony analysis based on the alignment of the L TOSV segment. The tree showed that the Tunisian sample sequences were clustered in four groups. One group included 7 sequences - 5 derived from the CSF sample and 2 from sandly pools - showed that significant similarities exist among the TOSV-isolated sequences described in France [GenBank: KC700343, DQ975233], in Italy [GenBank: X68414], and in Tunisia [GenBank: JX867537]. A second group (18 sequences) shows significant similarities with the TOSV Morocco isolate [GenBank: JN832571]. The third group contains a single sequence [DDJB: AB905362] derived from the sandfly pool and shows significant similarities with the Punique sequences described in Tunisia [GenBank: JF920133]. The fourth group contains a single sequence [DDJB: AB905361] derived from a SFC sample [DDJB: AB905361] and shows similarities with the SFSV isolated in Algeria (Figure 2).

## Discussion

The clinical manifestations of the TOSV infection are the same as those provoked by several other neurotropic viral agents, and require a rapid, sensitive laboratory diagnosis for confirmation. Serological techniques such as ELISA are useful for assaying specific IgM as a marker of an acute infection. In this study, a serological investigation for the TOSV infection was performed (in house-ELISA) on 263 samples that included sera (n = 191) and cerebrospinal fluids (n = 72) collected from patients with AMM from different regions of Tunisia. TOSV infection was recorded in 12.1% of the patients affected by meningitis during the summer, since anti-TOSV IgM were detected. IgG antibodies against TOSV were detected in 78.1% of the IgM-positive cases. The remaining 21.9% of IgM positive but IgG negative cases could reflect a very early phase of infection. Similar results were observed in Turkey where IgG and IgM antibodies were reactive with TOSV in 10.4% and 8.2% of the sera respectively, while only 4% were reactive for both IgG and IgM [28]. The detection of IgM, IgG or both, can be achieved in cells infected with TOSV. However, it is known that cross-reactivity exists between members of the Phlebovirus genus and specifically between TOSV and other serotypes of the Sandfly fever Naples serogroup [29],[30]. The interpretation of serological assay results in the Mediterranean region, which is endemic for several phleboviruses, may therefore be more difficult because of frequent recent infections and/or reinfections that boost immunity. However, ELISA or IFA tests can be used for screening purposes, but equivocal evaluations might require retesting and/or confirmation [31]. Despite the lack of confirmation by a seroneutralisation test, a total of 32 patients with IgM positive and with evocative symptoms are considered in this study to be probable cases of acute TOSV infection.

The overall 12.1% TOSV seroprevalence rate is quite similar to the rate observed in Tunisia by Bahri et al. [21], who attributed 10% of seasonal meningitis cases between 2003 and 2009 to TOSV. This rate resembles that recorded in Ankara, Turkey (11.2%) in patients with aseptic meningoencephalitis [32]. However, this rate is higher than that reported in Portugal (4.2%) in patients with neurological symptoms [33]. Analyzing healthy people from different bioclimatic zones of Tunisia [22] showed a 9.5% seroprevalence for TOSV. Recently, Sakhria et al. [34] recorded that the TOSV seroprevalence in Tunisia varied from 17.2% to 59.4% depending on the region. These observations emphasize the presence of asymptomatic infections related to TOSV in exposed individuals in Tunisia. The data confirm that TOSV infections can produce mild influenza-like or subclinical infections, since TOSV causes severe illness involving the central nervous system in only a few patients [8]. Several serological surveys have shown that TOSV is endemic in the Mediterranean Basin and that its seroprevalence varies with the region: 80% in Italy, 17.8% in Turkey and 25% in Spain [3],[18],[28].

Despite the limitation of serological tests, viral RNA detection has been established as a powerful tool and an effective alternative for identifying acute TOSV infections [3],[31]. This study indicates that among the CSF (n = 140) samples analyzed by RT-PCR, 12.8% tested positive for TOSV. Finding 18 cases of acute aseptic meningitis due to TOSV among patients from different regions in Tunisia confirms, for the first time, that TOSV is involved in this neurological disease in North Africa. The incidence of involvement resembles that observed in other Mediterranean Basin countries [32],[34]-[36].

With respect to the samples tested by serological and molecular techniques (72 of 140), the discrepancies in the results observed using these two diagnostic methods can be explained either by assays' different sensitivities or by the timing of drawing CSF samples. Similar finding were reported in Anatolia, Turkey where TOSV IgM was identified via indirect immunofluorescence test in only four of the 16 TOSV RNA positive samples [37].

The PCR amplicons were directly sequenced to confirm the TOSV genome and, possibly, to identify the viral strain circulating in Tunisia. However, due to the short sequences obtained with the primers Atos2 and NPhlebo2+ (126bp), we could not identify the strain of TOSV precisely.

Nevertheless, the sequences obtained correspond to those described in Morocco [20], France [15],[38] and Tunisia [23]. In Morocco, only the lineage B was described [20] whereas in France, the Italian lineage A and the Spanish lineage B co-circulate [38].

In this study, using RT-PCR, we also identified the presence of SFSV in the CSF sample of one febrile patient with meningoencephalitis from the Sousse region, situated in the lower semi-arid bioclimatic zone. SFSV have infrequently been implicated in central nervous system infections. However, the presence of symptoms and the detection of viral RNA confirmed that the Sousse patient suffered from an acute infection with SFSV from which he completely recovered. Similar cases of SFSV infection were described in a patient returning from vacation in Tunisia who was suffering from fever, headache, rigor, nausea, joint pains and a maculopapular [39]. In addition, sporadic cases from Turkey caused by this virus or a SFS-like virus were described [30],[40].

In earlier serological studies conducted in Tunisia, neutralized antibodies against SFSV were detected in 1.3% of tested sera [41]. By using the Hemagglutination Inhibition Test, 31% of sera collected from rodents, insectivores, and chiropters in Tunisia were positive for SFSV antibodies [42]. In addition, a new Sicilian-like virus provisionally named the Punique virus was detected in Laroussius flies in Utique, a city in northern Tunisia, although no isolation was obtained [43].

The sandfly fauna of Tunisia includes 17 species [44] whose density and diversity vary by bioclimatic zones. Of these, the dominant *P. papatasi, P. perniciosus* and *P. perfiliewi* are widespread in Tunisia where they transmit leishmaniasis to dogs and humans [45]. Our results show that they may also transmit TOSV. Indeed, RT-PCR performed on pools of six species of Phlebotominae trapped in northern Tunisia near the habitats of some TOSV-positive patients showed that only *P. perniciosus* and *P. perfiliewi* are TOSV-positive. This result correlates with previous work demonstrating that these species are the dominant arthropod TOSV vectors in Italy, Morocco and Spain [2],[20],[46]. In addition, during the course of this study, a peak of TOSV-related meningitis and encephalitis occurred during the warm season, which is the period of maximum activity of these sandfly vectors [47].

In the effort to isolate the virus from Phlebotomine pools by viral cultures, we isolated only the Punique virus from one pool of *P. perniciosus* collected in Medjez el Bab, located in the Beja governorate of Tunisia's upper semi arid bioclimatic zone. Recently described in Tunisia by Zhioua et al. [43], this phlebovirus is closely related to the Massilia virus but is largely different from other viruses (Naples, Toscana, Tehran, and Algeria viruses) in the SFV complex. The public health significance of the Punique virus is unknown at this time. However, given that its presence in Tunisia was confirmed despite any correlation with clinical manifestations, this new viral agent warrants further extensive research.

## Conclusions

Several unexplained cases of meningitis recorded every summer in Tunisian hospitals are confirmed as being due to TOSV. A sporadic case is attributed to SFSV. Indeed, the RNA of these viruses was detected in CSF from patients with neurological symptoms and in sandfly vectors (*P. perniciosus* and *P. perfiliewi*)*.* Global changes, including climate change, land use changes and mainly livestock development, may influence the density and future distribution of sandfly species. This may, in turn, have implications for the epidemiology of sandfly-borne Phleboviruses and create new risk areas and potential at-risk human populations. Consequently, surveillance programs should be designed to prevent the spread of sandflies in Tunisia.

TOSV should be considered an emergent viral pathogen that threatens healthy populations in Tunisia by causing seasonal aseptic meningitis. Clinicians should be aware of the role of TOSV and other Phleboviruses in cases of meningitis and RT-PCR should be considered a useful tool for their identification in clinical samples.

### Availability of supporting data

Links to accession numbers in the DDBJ

AB904897

http://getentry.ddbj.nig.ac.jp/getentry/na/AB904897/?format=flatfile&filetype=html&trace=true&show_suppressed=false&limit=10

AB904898

http://getentry.ddbj.nig.ac.jp/getentry/na/AB904898/?format=flatfile&filetype=html&trace=true&show_suppressed=false&limit=10

AB904899

http://getentry.ddbj.nig.ac.jp/getentry/na/AB904899/?format=flatfile&filetype=html&trace=true&show_suppressed=false&limit=10

AB904900

http://getentry.ddbj.nig.ac.jp/getentry/na/AB904900/?format=flatfile&filetype=html&trace=true&show_suppressed=false&limit=10

AB904901

http://getentry.ddbj.nig.ac.jp/getentry/na/AB904901/?format=flatfile&filetype=html&trace=true&show_suppressed=false&limit=10

AB904902

http://getentry.ddbj.nig.ac.jp/getentry/na/AB904902/?format=flatfile&filetype=html&trace=true&show_suppressed=false&limit=10

AB904903

http://getentry.ddbj.nig.ac.jp/getentry/na/AB904903/?format=flatfile&filetype=html&trace=true&show_suppressed=false&limit=10

AB904904

http://getentry.ddbj.nig.ac.jp/getentry/na/AB904904/?format=flatfile&filetype=html&trace=true&show_suppressed=false&limit=10

AB904905

http://getentry.ddbj.nig.ac.jp/getentry/na/AB904905/?format=flatfile&filetype=html&trace=true&show_suppressed=false&limit=10

AB904906

http://getentry.ddbj.nig.ac.jp/getentry/na/AB904906/?format=flatfile&filetype=html&trace=true&show_suppressed=false&limit=10

AB904907

http://getentry.ddbj.nig.ac.jp/getentry/na/AB904907/?format=flatfile&filetype=html&trace=true&show_suppressed=false&limit=10

AB904908

http://getentry.ddbj.nig.ac.jp/getentry/na/AB904908/?format=flatfile&filetype=html&trace=true&show_suppressed=false&limit=10

AB904909

http://getentry.ddbj.nig.ac.jp/getentry/na/AB904909/?format=flatfile&filetype=html&trace=true&show_suppressed=false&limit=10

AB904910

http://getentry.ddbj.nig.ac.jp/getentry/na/AB904910/?format=flatfile&filetype=html&trace=true&show_suppressed=false&limit=10

AB904911

http://getentry.ddbj.nig.ac.jp/getentry/na/AB904911/?format=flatfile&filetype=html&trace=true&show_suppressed=false&limit=10

AB904912

http://getentry.ddbj.nig.ac.jp/getentry/na/AB904912/?format=flatfile&filetype=html&trace=true&show_suppressed=false&limit=10

AB904913

http://getentry.ddbj.nig.ac.jp/getentry/na/AB904913/?format=flatfile&filetype=html&trace=true&show_suppressed=false&limit=10

AB904914

http://getentry.ddbj.nig.ac.jp/getentry/na/AB904914/?format=flatfile&filetype=html&trace=true&show_suppressed=false&limit=10

AB904915

http://getentry.ddbj.nig.ac.jp/getentry/na/AB904915/?format=flatfile&filetype=html&trace=true&show_suppressed=false&limit=10

AB904916

http://getentry.ddbj.nig.ac.jp/getentry/na/AB904916/?format=flatfile&filetype=html&trace=true&show_suppressed=false&limit=10

AB904917

http://getentry.ddbj.nig.ac.jp/getentry/na/AB904917/?format=flatfile&filetype=html&trace=true&show_suppressed=false&limit=10

AB904918

http://getentry.ddbj.nig.ac.jp/getentry/na/AB904918/?format=flatfile&filetype=html&trace=true&show_suppressed=false&limit=10

AB904919

http://getentry.ddbj.nig.ac.jp/getentry/na/AB904919/?format=flatfile&filetype=html&trace=true&show_suppressed=false&limit=10

AB904920

http://getentry.ddbj.nig.ac.jp/getentry/na/AB904920/?format=flatfile&filetype=html&trace=true&show_suppressed=false&limit=10

AB904921

http://getentry.ddbj.nig.ac.jp/getentry/na/AB904921/?format=flatfile&filetype=html&trace=true&show_suppressed=false&limit=10

AB904922

http://getentry.ddbj.nig.ac.jp/getentry/na/AB904922/?format=flatfile&filetype=html&trace=true&show_suppressed=false&limit=10

AB904923

http://getentry.ddbj.nig.ac.jp/getentry/na/AB904908/?format=flatfile&filetype=html&trace=true&show_suppressed=false&limit=10

AB904924

http://getentry.ddbj.nig.ac.jp/getentry/na/AB904908/?format=flatfile&filetype=html&trace=true&show_suppressed=false&limit=10

AB905360

http://getentry.ddbj.nig.ac.jp/getentry/na/AB905360/?format=flatfile&filetype=html&trace=true&show_suppressed=false&limit=10

AB905361

http://getentry.ddbj.nig.ac.jp/getentry/na/AB905361/?format=flatfile&filetype=html&trace=true&show_suppressed=false&limit=10

AB905362

http://getentry.ddbj.nig.ac.jp/getentry/na/AB905362/?format=flatfile&filetype=html&trace=true&show_suppressed=false&limit=10

## Authors' contributions

OF: collected samples, performed molecular and data analysis and drafted the manuscript; YM and GGS: participated to molecular and data analysis; LA, NH and HT: coordinated human sample collection and collected clinical data; MGC and AB: supervised the study and reviewed the manuscript. All authors read and approved the final manuscript.

## Authors’ original submitted files for images

Below are the links to the authors’ original submitted files for images. Authors’ original file for figure 1 Authors’ original file for figure 2
