# Supplementary figures and images for: Serological and molecular detection of Toscana and other Phleboviruses in patients and sandflies in Tunisia
Source: BMC Infect Dis. 2014 Nov 15;14:598. doi: 10.1186/s12879-014-0598-9 (PMC4236749; doi:10.1186/s12879-014-0598-9)

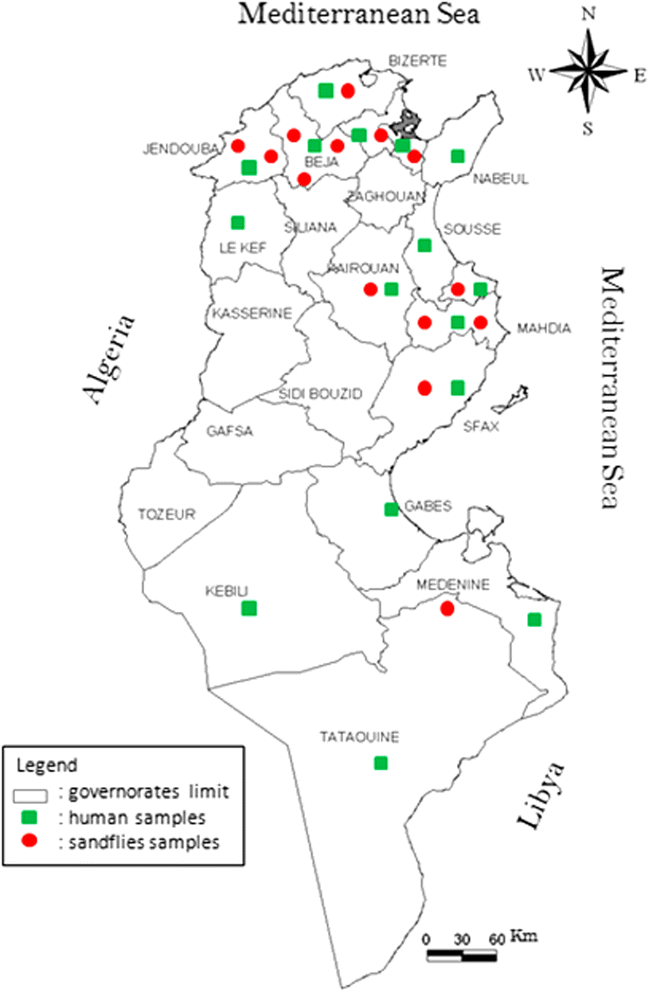

Supplement: Supplementary file 1 — Authors’ original file for figure 1 [file 12879_2014_598_MOESM1_ESM.gif]

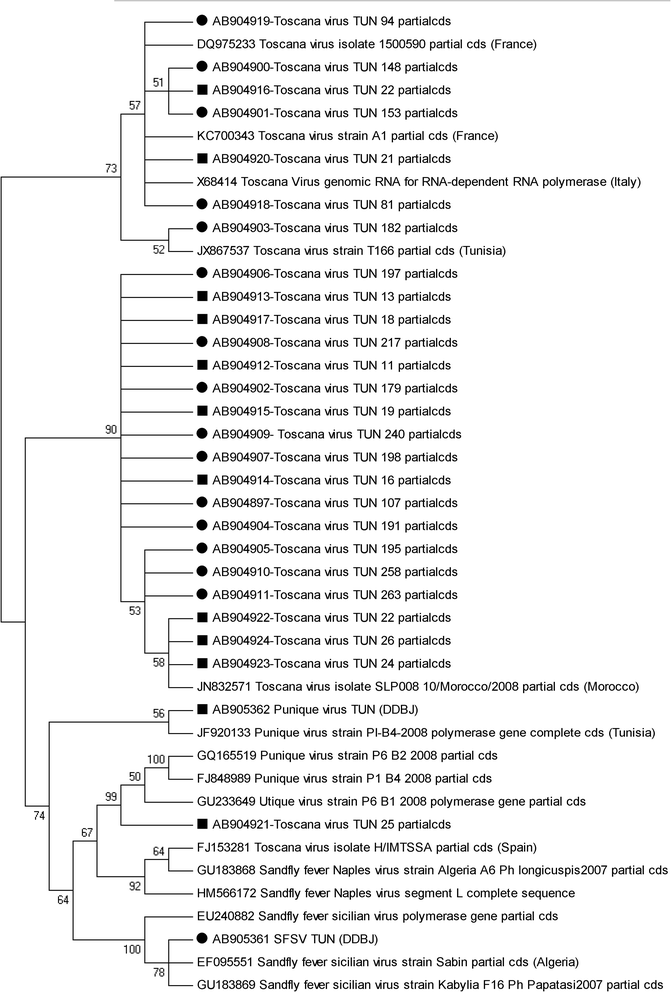

Supplement: Supplementary file 2 — Authors’ original file for figure 2 [file 12879_2014_598_MOESM2_ESM.gif]
